# Effectiveness of various interventions for non-traumatic osteonecrosis: a pairwise and network meta-analysis

**DOI:** 10.3389/fendo.2024.1428125

**Published:** 2024-08-21

**Authors:** Shaoyang Zhai, Rui Wu, Jie Zhao, Wang Huang, Weiwei Hu, Weichen Huang

**Affiliations:** ^1^ Orthopedic Injury College, Guizhou University of Traditional Chinese Medicine, Guiyang, Guizhou, China; ^2^ Joint Orthopedics, The Second Affiliated Hospital of Guizhou University of Traditional Chinese Medicine, Guiyang, Guizhou, China

**Keywords:** meta-analysis, osteonecrosis of the femoral head (ONFH), extracorporeal shock wave therapy (ESWT), core decompression (CD), multiple drilling decompression (MDD), vascularized or non-vascularized bone grafting surgery/resection (VGF), free vascularized fibular grafting (FVFG)

## Abstract

**Background:**

Osteonecrosis of the femoral head (ONFH) is acknowledged as a prevalent, challenging orthopedic condition for patients.

**Purpose:**

This study aimed to evaluate the efficacy of various interventions for non-traumatic ONFH and provide guidance for clinical decision-makers.

**Methods:**

We searched PubMed, Embase, Cochrane Library, and Web of Science databases from inception to February 2023 for relevant randomized controlled trials evaluating treatments for femoral head necrosis, without language restrictions. Quality evaluation was performed using the Cochrane risk-of-bias assessment tool, and analysis was performed using Stata 15.1.

**Results:**

Eleven randomized controlled trials were included in this study. The meta-analysis results revealed that CellTherapy [MD= -3.46, 95%CI= (-5.06, -1.85)], InjectableMed [MD= -3.68, 95%CI= (-6.11, -1.21)], ESWT [MD= -2.84, 95%CI= (-4.23, -1.45)], ESWT+InjectableMed [MD= -3.86, 95%CI= (-6.22, -1.53)] were significantly more effective in improving VAS pain score than CD+PTRI, as well as CD+BG+CellTherapy, and CD+BG. Furthermore, CD+BG+CellTherapy was better than CD+BG [MD= -0.97, 95%CI= (-1.71, -0.19)]. The SUCRA ranking for HHS score indicated that CellTherapy (77%) has the best effectiveness rate, followed by ESWT+InjectableMed (72.2%), ESWT (58.3%), InjectableMed (50%), CD+PTRI (31.4%), and CD+BG (11%). In terms of WOMAC and Lequesne scores, the meta-analysis showed no statistically significant differences between the experimental group CD+BG+CellTherapy and the control group CD+BG.

**Conclusion:**

CellTherapy and non-surgical ESWT combined with medication or CellTherapy have the best effect on ONFH. Surgical CD+BG combined with CellTherapy is more effective than CD+BG alone. ESWT+InjectableMed is recommended for short-term or acute onset patients, while ESWT is recommended for long-term patients.

**Systematic review registration:**

https://www.crd.york.ac.uk/PROSPERO, identifier CRD42024540122.

## Introduction

Osteonecrosis of the femoral head (ONFH) is a bone disease that can be classified into traumatic and non-traumatic categories. It is diagnosed through various methods, including clinical symptoms, imaging tests, radionuclide examinations, bone biopsy, and digital subtraction angiography. The Association Research Circulation Osseous Classification (ARCO) staging system, which was developed in 1991, is widely used to stage the disease (1). Non-traumatic ONFH, also known as ischemic or aseptic necrosis, is a common but difficult-to-treat bone disease caused by abnormal blood supply to the bone tissue. The exact pathophysiology of ONFH is not fully understood but may be caused by factors such as coagulation disorders, drugs, alcohol abuse, trauma, or genetics, which can lead to complications in the blood vessels of the femoral head. This can result in bone tissue death, structural remodeling, and collapse. ONFH is prevalent in many Asian countries and occurs in 10,000 to 20,000 adults each year in the United States. It accounts for 5 -18% of the 500,000 total hip arthroplasties performed annually in North America alone (2). Acute pain caused by femoral head necrosis usually occurs after early weight-bearing and can have a significant impact on the life quality, mental health, and personal and socio-economic well-being of the patient. Early intervention in the early stages of femoral head necrosis is necessary to prevent progressive deterioration. The main treatment strategies for ONFH include oxygen therapy, bisphosphonates, hyperbaric electrical stimulation, core decompression (CD), vascularized bone transplantation, and extracorporeal shockwave therapy (ESWT). With the development of new technologies, CellTherapy methods such as bone marrow mononuclear cell transplantation have also been studied in clinical practice. If the necrosis process cannot be controlled, total hip arthroplasty (THA) may eventually be necessary (3).

Currently, core decompression is the most generally used treatment method in the early stage of ONFH, and vascularized bone grafting is recommended in the early stage of ARCO III. Core decompression aims to reduce intraosseous pressure and may enhance intravascular growth, thereby relieving pain, delaying, or avoiding the need for total hip arthroplasty (4). However, it should be used with caution in the late stage of femoral head necrosis. Furthermore, as a surgical treatment option, CD may involve complications such as incomplete intervention, secondary surgery, or situations where the treatment cannot be accepted due to physical conditions and age (5). Extracorporeal shockwave therapy (ESWT) is a safe and effective non-invasive treatment method that can improve the healing process, although its mechanism of action is not yet fully understood. It is reported that its mechanism is similar to the nuclear cascade process triggered by mechanotransduction. In theory, mechanical energy can cause changes in the cell skeleton, which can lead to a response in the cell’s marrow (such as the release of mRNA), thereby affecting different cell structures such as intracellular vesicles, endoplasmic reticulum, and mitochondria. Therefore, enzyme reactions can improve the healing process (6). In 2018, a systematic review and network meta-analysis conducted by Ji Wang et al. (7), indicated that ESWT was the most effective intervention in improving the Harris Hip Score (HHS) for ONFH patients, and vascularized fibular grafting (VFG) showed a better effect in reducing treatment failure rates. However, the original literature included in their study were all retrospective studies. Therefore, given many newly-published randomized clinical trials (RCTs) on various interventions for ONFH, an updated network meta-analysis based on randomized controlled trials and pairwise meta-analysis of interventions that could not be networked were conducted to provide new evidence-based for clinical decision-makers. The purpose of this study is to comprehensively compare the efficacy of various interventions in the treatment of non-traumatic ONFH patients.

## Materials and methods

Our study was conducted in accordance with the Cochrane Handbook for the Systematic Review of Interventions and the Preferred Reporting Items for Systematic Review and Meta-Analyses (8).

### Inclusion criteria

#### Research type

Randomized controlled trials.

#### Research subjects

1. Age 18 or older.
2. Clinically diagnosed as non-traumatic ONFH.
3. Have met the application indications of ARCO or Ficat I to III stages of femoral head osteonecrosis.

### Intervention measures

Free fibula graft (FGF), free vascularized fibula graft (FVFG), extracorporeal shock wave therapy (ESWT), autogenous iliac bone graft (ABG), core decompression (CD), multiple drilling decompression (MDD), and intraosseous drug injection therapy.

### Outcome indicators

The main outcome measures are visual analogue scale (VAS) and HHS. The secondary outcome indicators are Lequesne and WOMAC.

### Exclusion criteria

1. Meta-analysis, review, systematic review, case report, animal experiment, non-comparable control, non-research disease, non-English literature, inconsistent intervention, letter, guideline, conference paper.
2. Pathological mechanism, non-randomized controlled experiment, single-arm study.
3. Literature that is not available in full text.

### Retrieval strategies

We adopted mesh terms and free keywords in the search strategy, according to the PICOS principle.

1. Population (P): Patients aged 18 or older clinically diagnosed with femoral head osteonecrosis and have met the application indications for non-traumatic femoral head osteonecrosis ARCO or Ficat stage I-III.
2. Intervention (I)/Comparison (C): The implementing of free fibular graft (FGF), extracorporeal shock wave therapy (ESWT), free vascularized fibular graft (FVFG), autogenous bone graft (ABG), core decompression (CD), multiple drilling decompression (MDD), or intraosseous drug injection therapy.
3. Outcome (O): VAS, HHS, Lequsne, and WOMAC.
4. Study design (S): RCTs.

A literature search was conducted by two independent researchers on four databases: EMBASE, PubMed, Cochrane Library, and Web of Science. The search utilized the following English terms: “osteonecrosis,” “free fibula graft (FGF),” “free vascularized fibula graft (FVFG),” “autogenous iliac bone graft (ABG),” “core decompression (CD),” “extracorporeal shock wave therapy (ESWT),” “multiple drilling decompression (MDD),” and “intraosseous drug injection.” The search period for all databases was from inception to February 2023, and there was no language restriction during the search. The detailed search strategy is presented in **Appendix 1**.

### Literature screening and data extraction

According to the inclusion and exclusion criteria, two researchers conducted a literature search. All identified literature was managed using Endnote software version X9. The researchers imported the literature into Endnote X9, removed duplicates, and screened the titles or abstracts to identify studies that met the criteria for this systematic review. The full-text articles of the selected studies were downloaded and read to identify eligible articles. The basic information of the included studies was extracted and cross-checked, and the units of measurement were standardized. In case of differences in opinions, the researchers discussed the issue with a third researcher to reach a decision. The extracted information mostly included the title, first author, publication year, country, study design, sample size, patient age, intervention methods, follow-up period, and outcome indicators.

### Risk of bias assessment

Two independent researchers assessed the risk of bias in eligible studies using the Cochrane risk-of-bias tool for randomized trials (RoB 2), which is recommended in the Cochrane Handbook for Systematic Reviews of Interventions version 6.3. The results of the assessments were cross validated to ensure consistency. The evaluation included seven aspects: (1) allocation concealment, (2) random sequence generation, (3) completeness of outcome data, (4) blinding of participants, personnel, and outcome assessors, (5) blinding of outcome assessment, (6) selective reporting of results, and (7) other biases. Each aspect was classified into three levels: low risk, unclear, and high risk. The risk of bias graph and summary figure were drawn using Review Manager (RevMan) version 5.4 software.

### Statistical methods

In this study, R software version 4.1.3, along with the gemtc package version 1.0-1, and JAGS software was adopted to perform a network meta-analysis based on the Bayesian framework, utilizing the Markov Chain Monte Carlo (MCMC) method. We employed four Markov chains for simulation analysis, using an initial value of 2.5 and a refining iteration step of 1. For annealing, we used 5000 pre-simulation iterations, followed by 20000 iterations to achieve model convergence. To assess model fitting and global consistency, the Deviance Information Criterion (DIC) was used. The consistency model was used for modeling if the absolute value of the DIC for consistency and inconsistency was less than five. In cases where a closed-loop network was present, the node-splitting method was used to analyze local consistency.

To determine the effect size, the risk ratio (RR) was applied for dichotomous data, and the weighted mean difference (WMD) for continuous data. A P-value less than 0.05 or a 95% credible interval (95% CI) (with dichotomous data not including one, and continuous data not including zero) was considered as the standard for statistical significance.

For interventions that could not form a network, we conducted pairwise meta-analysis using Stata 15.1 software. Continuous variables were pooled using the standardized mean difference (SMD) and the 95% confidence interval (CI) was calculated. Moreover, heterogeneity was assessed using the Q statistic and I² test. If the heterogeneity among studies was acceptable (P > 0.1 and I² ≤ 50%), the fixed-effect model was used for meta-analysis. However, if the heterogeneity among studies was significant (P ≤ 0.1 or I² > 50%), the random-effects model was used for meta-analysis. Furthermore, the “metabias” command was used to identify publication bias in the included studies, results with a P-value less than 0.05 were considered statistically significant.

The analysis yielded a range of results, including a network evidence relationship diagram, forest plot, ranking probability bar chart, and league table for each outcome indicator. The surface under the cumulative ranking curve (SUCRA) was used as an index for the cumulative ranking probability to rank the interventions based on the SUCRA value, with higher values indicating better interventions. All the analysis processes for this pairwise and network meta-analysis were carried out in Stata 15.1 and R software version 4.1.3.

## Results

### Literature search process

**Figure 1:**
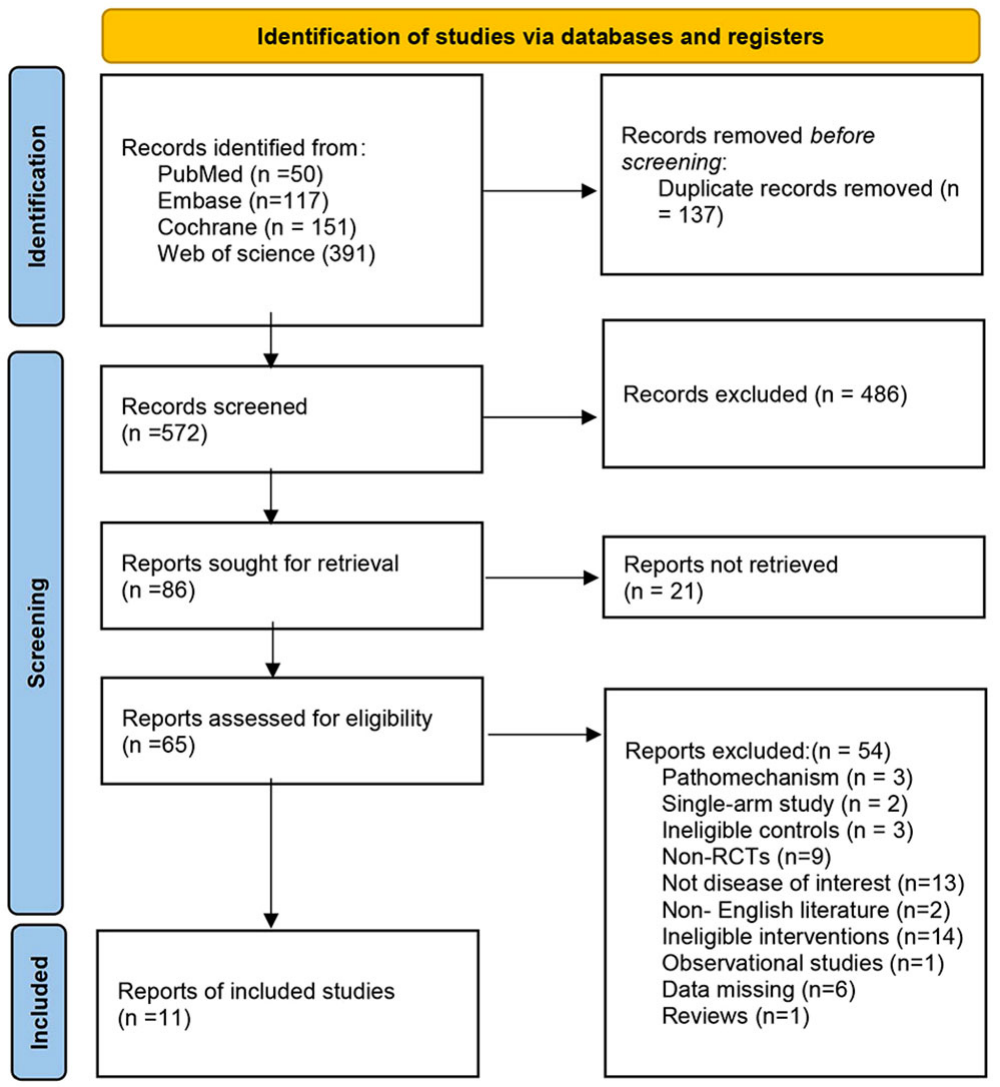
The literature search process.

A systematic literature search was carried out, and a total of 710 articles were initially identified. After removing duplicates and screening for relevance, 11 articles were included in the final analysis. The literature search process is shown in **Figure 1**.

### Basic characteristics of included literature

The 11 included articles involved a total of 685 study subjects, with 343 subjects in the experimental group and 342 subjects in the control group. All the RCT studies on intervention indicators were published in English. Further details regarding the basic characteristics of the included literature are provided in **Table 1**.

**Table 1:** Basic characteristics of included literature.

Author, Year	Country	Study Type	Treatment		Sample Size (E/C)	Stage Classification	Follow Up time	Outcome indicators	
			Experiment group	Control group				Network	Pairwise
XuYang2022	China	RCT	ESWT +InjectableMED (ALD)	InjectableMED (ALD)	75/78	ARCOIV stage(ONFH)	12 mon	①②	–
Lijun Shi2022	China	RCT	ESWT +InjectableMED (TCM&ALD)	InjectableMED (TCM&ALD)	73/70	ARCOI, II, IIIa stage	12 mon	①②	–
Ching-Jen Wang2007	China	RCT	ESWT +InjectableMED (ALD)	ESWT	23/25	ARCOI, II, III stage	12mon	①②	–
CHING-JEN WANG2005	China	RCT	CD+BG	ESWT	25/23	ARCOI, II, III stage	24mon	①②	–
Keyun Peng2020	China	RCT	CD+BG	CD+PTRI	30/30	ARCOI, II stage	12mon	①②	–
Yuanchen Ma2014	China	RCT	CD + BG + CellTherapy (BBC)	CD+BG	21/18	FicatI, II, III stage	24mon	①	③④
Shuo Luan2022	China	RCT	CellTherapy (PRP)	ESWT	30/30	ARCOI, II, III stage	12mon	①②	–
Mengyuan Li2020	China	RCT	CD + BG + CellTherapy (BBC)	CD + BG	17/14	FicatII, III stage	120mon	①	③④
Jean-PhilippeHauzeur2017	Belgium	RCT	CD + CellTherapy (BMAC)	CD	19/19	ARCOIII stage	24mon	–	①
Wojciech Pepke2016	Germany	RCT	CD + CellTherapy (BMAC)	CD	11/14	ARCOII stage	24mon	–	①②
Aditya K. Aggarwa l2020	India	RCT	CD + PRP	CD	19/21	Fica and ArletI, II stage	24mon	–	②

①VAS; ②HHS; ③WOMAC; ④Lequsne. ESWT, extracorporeal shock wave therapy; ALD, Alendronate sodium tablets; TCM, Chinese herbal Fufang Xian Ling Gu Bao; CD, core decompression; BG, bone grafting; PRP, platelet-rich plasma; BMAC, bone marrow aspirate concentrate; BBC, bone-marrow buffy coat; PTRI, porous tantalum rod implantation.

### Quality evaluation

**Figure 2:**
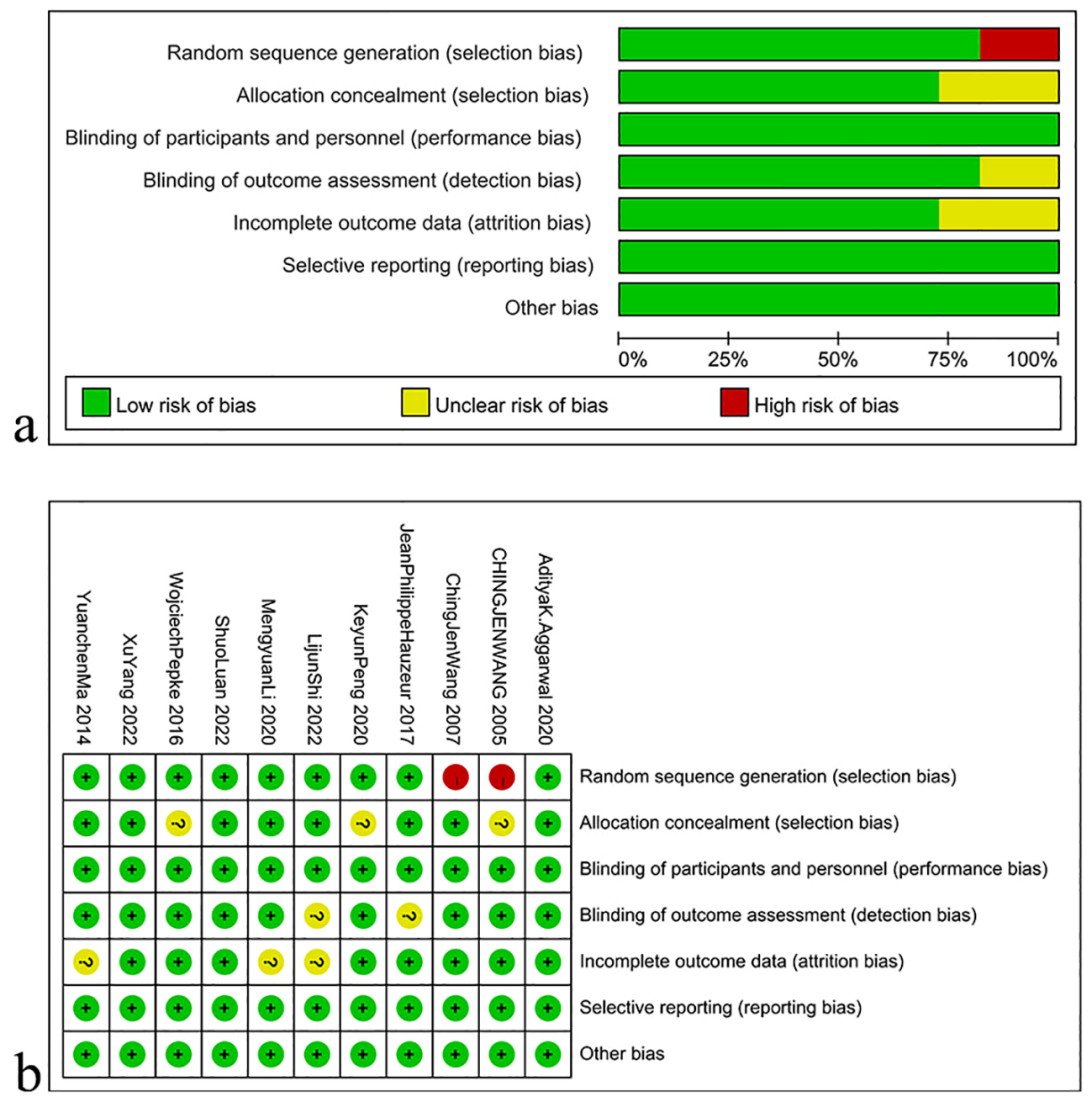
The bias risk assessment of the included literature. **(A)** Risk of bias graph; **(B)** Risk of bias summary.

The assessment of study quality conducted on the included literature demonstrated that two studies did not report the use of randomization, allocation concealment methods were not mentioned in two other studies, and blinding was not reported in two studies. Furthermore, seven studies did not report whether outcome assessors were blinded. Nevertheless, no risk of attrition bias, reporting bias, or any other types of biases was found in any of the studies. The bias risk assessment of the included literature is depicted in **Figure 2**.

### Network meta-analysis of included studies

#### VAS pain score network meta-analysis

**Figure 3:**
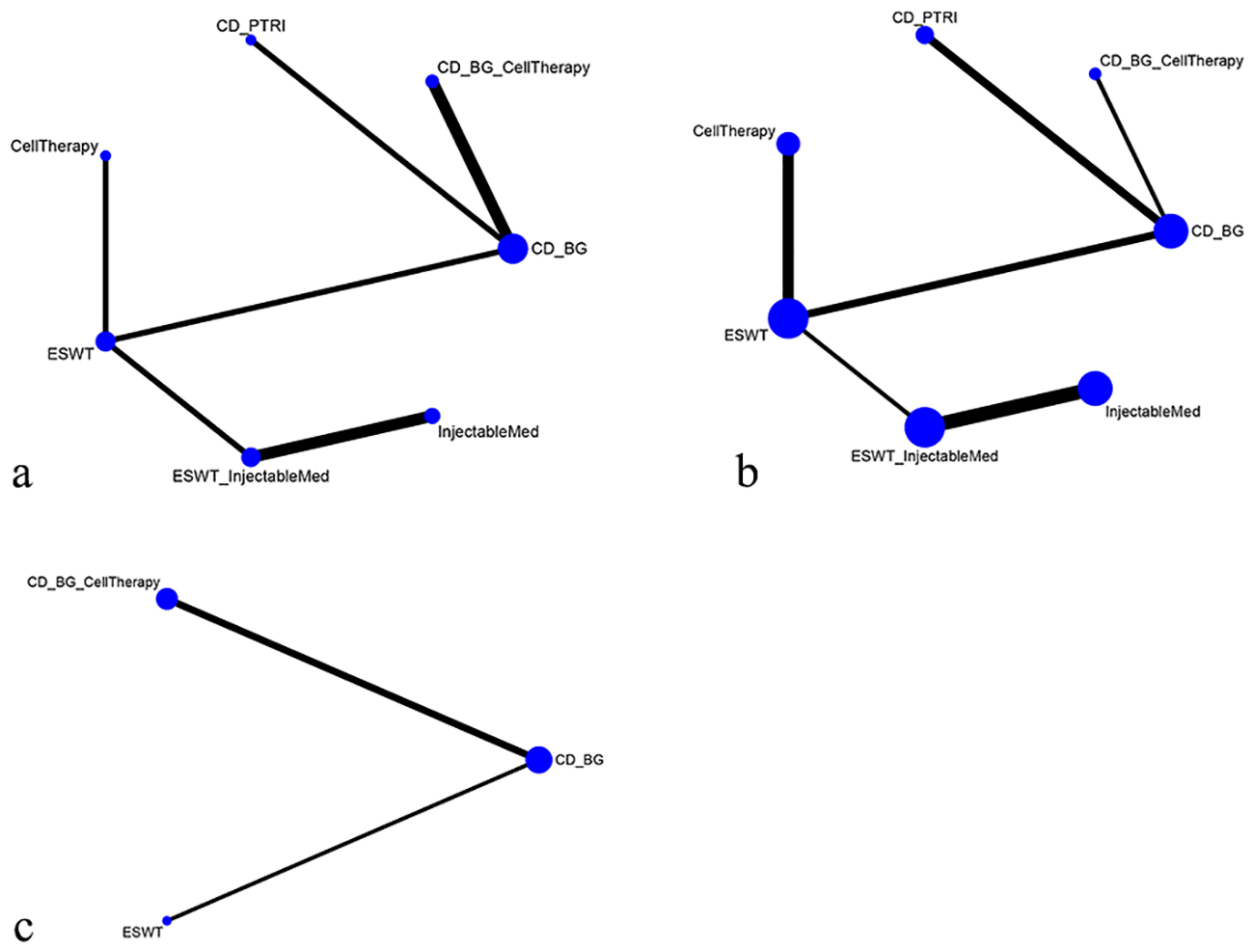
**(A)** VAS network plot; **(B)** VAS network plot short-term effect; **(C)** VAS network plot long-term effect.

This network meta-analysis included eight randomized controlled trials (9–16), involving 642 patients across seven interventions: CellTherapy, ESWT, ESWT+InjectableMed, InjectableMed, CD+PTRI, CD+BG+ CellTherapy, and CD+BG. The league table results showed that the CellTherapy intervention has a better pain relief effect than CD+PTRI [MD= -3.46, 95% CI= (-5.06, -1.85)], CD+BG+ CellTherapy [MD= -2.84, 95% CI= (-4.41, -1.31)], and CD+BG [MD= -3.81, 95% CI= (-5.12, -2.47)]. However, the CD+BG+CellTherapy intervention has a better pain relief effect than CD+BG alone [MD= -0.97, 95% CI= (-1.71, -0.19)]. Furthermore, the ESWT intervention has a better pain relief effect than CD+PTRI [MD= -2.84, 95% CI= (-4.23, -1.45)], CD+BG+ CellTherapy [MD= -2.22, 95% CI= (-3.56, -0.91)], and CD+BG [MD= -3.19, 95% CI= (-4.28, -2.10)]. However, the ESWT+ InjectableMed intervention has a better pain relief effect than CD+PTRI [MD= -3.86, 95% CI= (-6.22, -1.53)], CD+BG+ CellTherapy [MD= -3.25, 95% CI= (-5.57, -0.97)], and CD+BG [MD= -4.22, 95% CI= (-6.40, -2.05)]. Additionally, the InjectableMed intervention has a better pain relief effect than CD+PTRI [MD= -3.68, 95% CI= (-6.11, -1.21)], CD+BG+ CellTherapy [MD= -3.06, 95% CI= (-5.48, -0.64)], and CD+BG [MD= -4.03, 95% CI= (-6.31, -1.72)]. There were no significant statistical differences among the remaining interventions. VAS network plot is shown in **Figure 3A**.

In order to investigate the impact of intervention durations on treatment effects, we conducted subgroup analyses based on short-term and long-term effects (intervention duration less or greater than 12 months), as shown in **Figures 3B, C**. The subgroup network meta-analysis found that, in the short-term intervention duration, CD+PTRI [MD =3.32, 95%CI= (1.44, 5.21)] and CD+BG [MD= 3.67, 95%CI= (2.05, 5.30)] were less effective than CellTherapy in relieving pain; CD+PTRI [MD= 2.70, 95%CI= (1.02, 4.38)] and CD+BG [MD= 3.05, 95%CI= (1.67, 4.44)] were less effective than ESWT; CD+PTRI [MD= 3.73, 95%CI= (1.13, 6.31)] and CD+BG [MD= 4.08, 95%CI= (1.66, 6.49)] were less effective than ESWT+InjectableMed; CD+PTRI [MD= 3.53, 95%CI= (0.79, 6.21)] and CD+BG [MD= 3.88, 95%CI= (1.32, 6.39)] were less effective than Injectable Med. In the long-term duration, CD+BG [MD= 3.49, 95%CI= (0.22, 6.78)] was less effective than ESWT in relieving pain. There were no significant differences between the other intervention measures. The network meta-analysis results of VAS pain score are summarized in **Table 2**.

**Table 2:** VAS pain score network meta-analysis results.

CD+BG	-0.97 (-1.71, -0.19)*	-0.35 (-1.23, 0.52)	-3.81 (-5.12, -2.47)*	-3.19 (-4.28, -2.10)*	-4.22 (-6.40, -2.05)*	-4.03 (-6.31, -1.72)*
1.45 (-0.16, 3.07)	CD+BG+ CellTherapy	0.62 (-0.56, 1.76)	-2.84 (-4.41, -1.31)*	-2.22 (-3.56, -0.91)*	-3.25 (-5.57, -0.97)*	-3.06 (-5.48, -0.64)*
0.82 (-0.86, 2.37)						
0.35 (-0.60, 1.30)	-1.10 (-2.97, 0.77)	CD+PTRI	-3.46 (-5.06, -1.85)*	-2.84 (-4.23, -1.45)*	-3.86 (-6.22, -1.53)*	-3.68 (-6.11, -1.21)*
-	-					
3.67 (2.05, 5.30)*	2.22 (-0.06, 4.52)	3.32 (1.44, 5.21)*	CellTherapy	0.62 (-0.18, 1.42)	-0.41 (-2.46, 1.63)	-0.22 (-2.38, 1.97)
**-**	-	**-**				
3.05 (1.67, 4.44)*	1.60 (-0.52, 3.72)	2.70 (1.02, 4.38)*	-0.62 (-1.48, 0.23)	ESWT	-1.03 (-2.91, 0.85)	-0.84 (-2.84, 1.21)
3.49 (0.22, 6.78)*	2.69 (-0.91, 6.39)	**-**	-			
4.08 (1.66, 6.49)*	2.63 (-0.27, 5.53)	3.73 (1.13, 6.31)*	0.41 (-1.75, 2.57)	1.03 (-0.95, 3.02)	ESWT+ InjectableMed	0.19 (-0.52, 0.95)
**-**	-	**-**	-	-		
3.88 (1.32, 6.39)*	2.43 (-0.59, 5.42)	3.53 (0.79, 6.21)*	0.21 (-2.11, 2.48)	0.83 (-1.32, 2.94)	-0.19 (-1.00, 0.55)	InjectableMed
-	-	-	-	-	-	

In the central diagonal, dark blue cells signify interventions. Cells below the interventions diagonal show efficacy: s.m.d. and 95% confidence intervals are reported. The light green cells indicate the efficacy of total intervention duration. The light-yellow cells indicate the efficacy within 12 months, while dark yellow indicate the efficacy after 12 months. Statistically significant results are in bold, with the star key* in the corresponding cells.

During the overall intervention period, ESWT+InjectableMed (87%) showed the highest effectiveness rate, followed by CellTherapy (78.3%), Injectab leMed (77.7%), ESWT (56.6%), CD+BG+ CellTherapy (31.1%), CD+PTRI (15.7%), and CD+BG (3.5%). However, in the short-term duration, ESWT+InjectableMed (86.2%) had the highest effectiveness rate, followed by CellTherapy (77.9%), InjectableMed (76.6%), ESWT (56%), CD+BG+CellTherapy (31.1%), CD+PTRI (15.2%), and CD+BG (4.2%). Meanwhile, ESWT had the highest efficacy followed by CD+BG+CellTherapy and CD+BG, in the long-term duration. The SUCRA of VAS is described in **Table 3**.

**Table 3:** Results of surface under cumulative ranking curve (SUCRA) of VAS.

Cell	SUCRA (%)		
	Total intervention period	less than 12 months	More than 12 months
CD+BG	3.5	4.2	6.7
CD+BG+ CellTherapy	31.1	33.9	46.9
CD+PTRI	15.7	15.2	–
CellTherapy	78.3	77.9	–
ESWT	56.6	56	96.4
ESWT+ InjectableMed	87	86.2	–
InjectableMed	77.7	76.6	–

### HHS network meta-analysis

**Figure 4:**
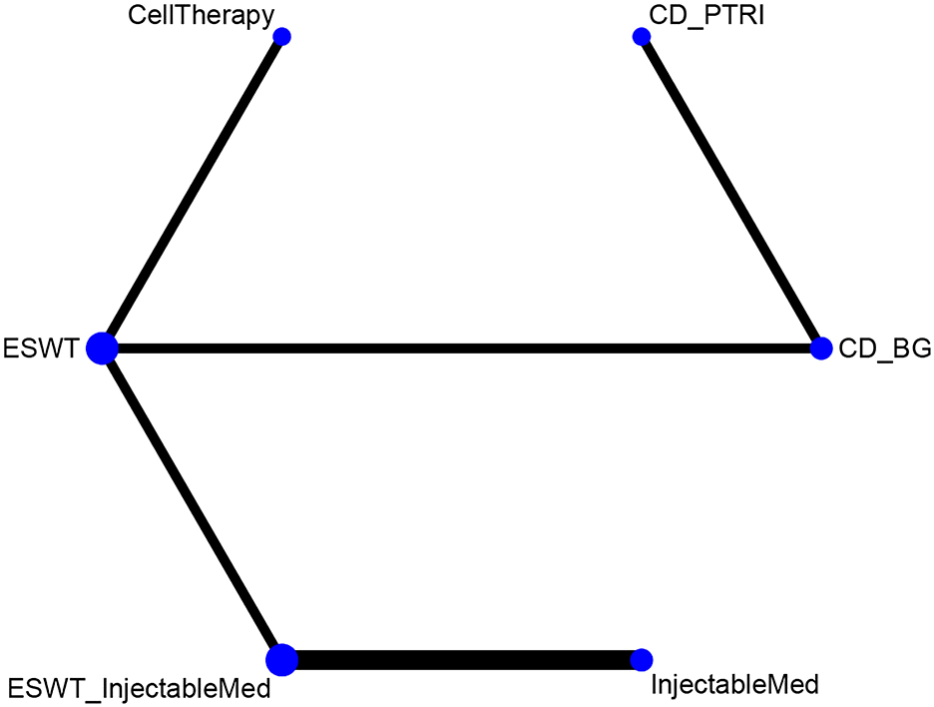
HHS network plot.

This network meta-analysis aims to compare the effectiveness of six intervention measures for HHS pain scores. The measures included CellTherapy, ESWT, InjectableMed, ESWT+InjectableMed, CD+PTRI, and CD+BG. The analysis involved six RCTs with a total of 512 patients (9–12, 14, 15). **Figure 4** illustrates HHS network analysis results. The league table results revealed no statistically significant differences between the pairwise comparisons of the intervention measures, as shown in **Table 4**. However, in terms of effectiveness ranking, CellTherapy (77%) was found to be the most effective intervention, followed by ESWT+ InjectableMed (72.2%), ESWT (58.3%), InjectableMed (50%), CD+PTRI (31.4%), and CD+BG (11%), as exhibited in **Table 5**.

**Table 4:** HHS network meta-analysis results.

CD+BG					
-5.90 (-26.12, 14.27)	CD+PTRI				
-21.47 (-50.21, 7.35)	-15.57 (-50.73, 19.56)	CellTherapy			
-16.06(-36.65, 4.39)	-10.16 (-38.99, 18.70)	5.37 (-14.94, 25.73)	ESWT		
-19.13 (-48.27, 9.94)	-13.25 (-48.47, 22.11)	2.32(-26.62, 31.00)	-3.05(-23.58, 17.46)	ESWT+ Injectable med	
-14.62 (-46.76, 18.08)	-8.73 (-46.52, 29.73)	6.86 (-25.23, 39.09)	1.473 (-23.37, 26.79)	4.51 (-9.47, 19.01)	Injectable med

In the central diagonal, dark blue cells signify interventions. Cells below the interventions diagonal show efficacy: s.m.d. and 95% confidence intervals are reported. The yellow cells indicate the efficacy of intervention.

**Table 5:** Results of surface under cumulative ranking curve (SUCRA) of HHS.

Cell	SUCRA (%)
CD+BG	11
CD+PTRI	31.4
CellTherapy	77
ESWT	58.3
ESWT+InjectableMed	72.2
InjectableMed	50

### Pairwise meta-analysis of included studies

### VAS meta-analysis

**Figure 5:**
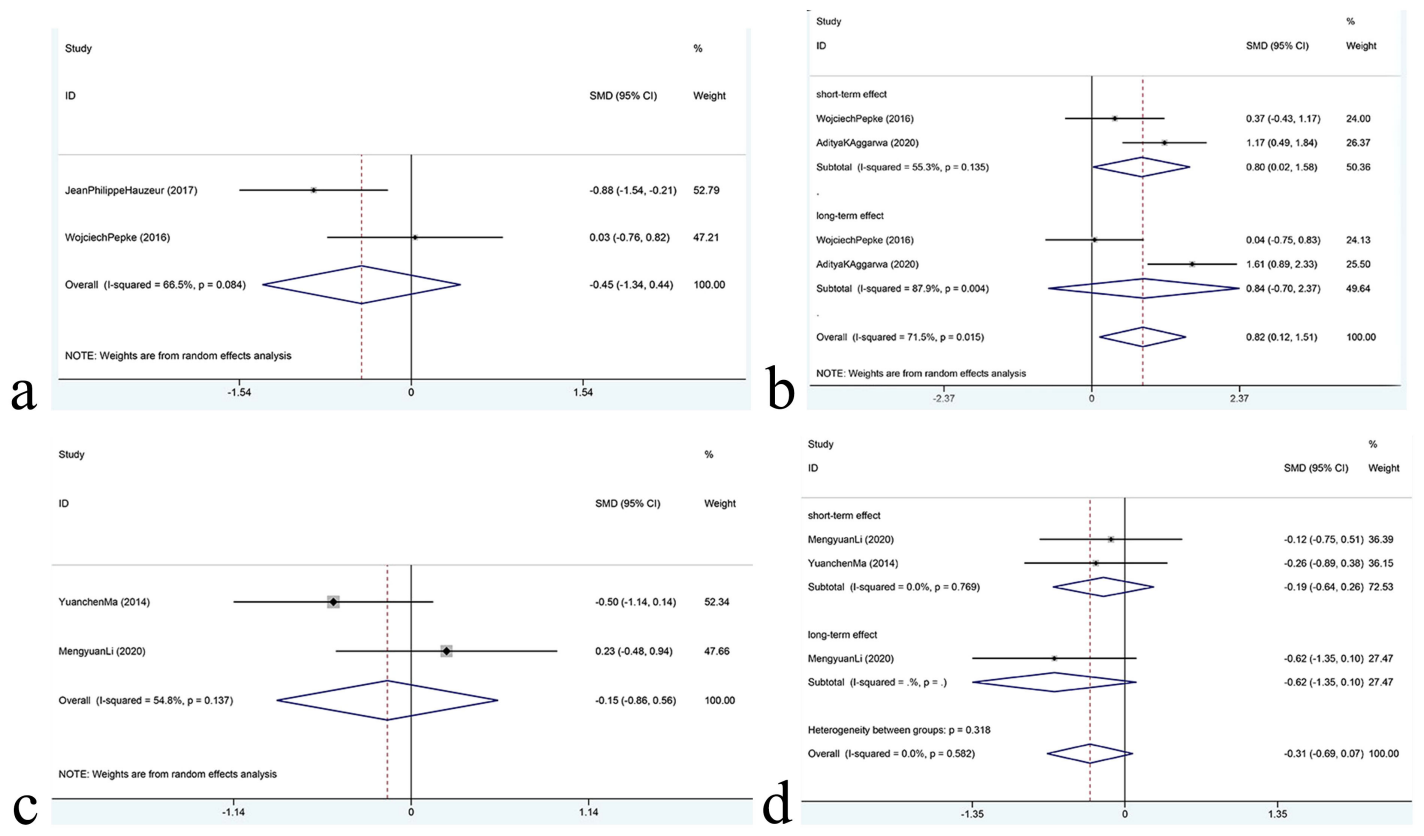
**(A)** The meta-analysis forest map for VAS; **(B)** The meta-analysis forest map for HHS; **(C)** The meta-analysis forest map for WOMAC; **(D)** The meta-analysis forest map for Lequsne.

This meta-analysis aims to investigate the intervention effects of CD+CellTherapy and CD alone on VAS scores of patients with osteonecrosis. Two RCTs (17, 18) were included in the study, with 30 cases in the experimental group and 33 cases in the control group. The random-effects model (I²=66.5%, P=0.084) was used to combine effect sizes. The analysis results showed no statistically significant difference in pain relief between CD+ CellTherapy and CD alone [SMD = -0.45, 95%CI= (-1.34, 0.44)]. The meta-analysis forest map for VAS is depicted in **Figure 5A**.

### HHS meta-analysis

Two randomized controlled trials (17, 19) investigated the effect of CD+CellTherapy versus CD alone on HHS scores of the improvement of patients with osteonecrosis, with 30 patients in the experimental group and 35 patients in the control group. A random-effects model (I²=71.5%, P=0.015) was used to combine effect sizes. The analysis showed that CD+CellTherapy exhibited a significant effect on improving HHS compared to CD alone [SMD= 0.82, 95%CI= (0.12, 1.51)]. Subgroup analysis was performed on the long-term and short-term effects of the intervention. The results showed that the short-term effect of CD+CellTherapy was significantly better than that of CD alone [SMD= 0.8, 95%CI= (0.02, 1.58)]. However, there was no statistically significant difference between the two groups in the comparison of long-term effects [SMD= 0.84, 95%CI= (-0.70, 2.37)]. The meta-analysis forest map for HHS is shown in **Figure 5B**.

### WOMAC meta-analysis

Two randomized controlled trials (17, 19) examined the effects of CD+BG+CellTherapy versus CD+BG on WOMAC scores in patients with osteonecrosis, with 38 participants in the experimental group and 32 in the control group. A random-effects model was used to combine effect sizes. The analysis showed no significant difference in the relief of WOMAC between the two groups [SMD= -0.15, 95%CI= (-0.86, 0.56)], as depicted in **Figure 5C**. The I² statistic was 54.8%, indicating moderate heterogeneity among the studies (P=0.137).

### Lequesne meta-analysis

Two randomized controlled trials (13, 16) investigated the effects of CD+BG+ CellTherapy versus CD+BG on Lequesne scores in patients with osteonecrosis, involving 38 cases in the experimental group and 32 cases in the control group. A fixed-effect model (I²=0.0%, P=0.582) was used to merge the effect sizes. The analysis result indicated no significant difference in the alleviation effect of Lequesne between CD+BG+CellTherapy and CD+BG [SMD= -0.31, 95%CI= (-0.69, 0.07)]. Subgroup analysis was conducted to analyze the short-term and long-term intervention effects, which revealed no statistical difference between the two groups for both short-term [SMD= -0.19, 95%CI= (-0.75, 0.51)] and long-term effects [SMD= -0.62, 95%CI= (-1.35, 0.10)]. The meta-analysis forest map for Lequsne is depicted in **Figure 5D**.

### Publication bias and inconsistency

**Figure 6:**
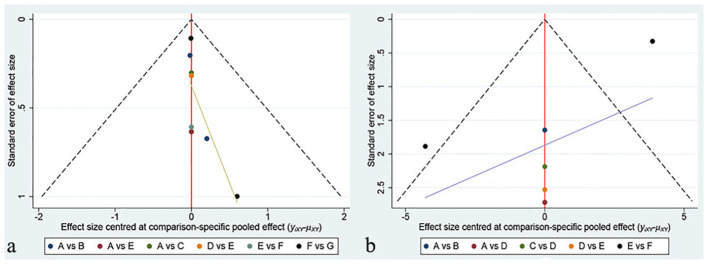
The funnel plots of **(A)** VAS and **(B)** HHS.

Funnel plots were created using Stata software to evaluate publication bias, with each color representing a different intervention comparison. Our network meta-analysis found no significant differences in the results for both the VAS and HHS indicators in the funnel plots, as displayed in **Figure 6**. Due to the limited number of studies included, we did not conduct publication bias tests for pairwise meta-analyses. Since our network diagram did not contain closed loops, inconsistency assessment was not applicable to our study.

## Discussion

The incidence of non-traumatic ONFH is on the rise, and it is uncertain whether this trend reflects a true increase or improved awareness and diagnostic capabilities. Roughly 10% of THAs performed in the United States each year are for ONFH (20). Although THA is the most effective treatment for end-stage ONFH, it is associated with complications related to the prosthetic joint and may yield unsatisfactory outcomes in younger or active patients. Non-joint-preserving surgeries are commonly used to treat ONFH patients (21). Biotechnological advances have led to research exploring the use of stem cells for ONFH treatment (22). Stem cells possess the capability to self-renew and segregate into various cell types, such as endothelial cells and osteoblasts, which mediate bone repair and vascular regeneration. Moreover, they release growth factors to endorse blood supply to the necrotic area through paracrine effects. Stem CellTherapy has emerged as a hip-preserving alternative for ONFH. Furthermore, studies have demonstrated the excellent effects of ESWT in treating non-traumatic ONFH, with few known side effects such as pain and mild bruising during the procedure. Serious complications are not expected if patients were treated according to recommended guidelines (6). CD has been utilized in early-stage ONFH with the intention of preventing femoral head collapse and potentially reversing disease progression. However, the outcomes of CD have been inconsistent, leading to questions about its effectiveness. More recently, traditional CD has been combined with the injection of BMAC to enhance results. Initial studies suggested that this additional cell therapy was effective (23, 24), but subsequent research found no significant differences in outcomes between CD with BMAC and CD alone. Both CD and BMAC therapy exhibited high rates of progression in large lesions. The effectiveness of BMAC remains a controversial topic that requires further investigation (25).

This study employed pairwise meta-analysis and network meta-analysis to synthesize direct and indirect evidence from 11 RCTs comparing the efficacy of various interventions for ONFH. Additionally, we examined the effect of follow-up time points on the results and performed subgroup analyses accordingly. Given the large number of intervention types, we grouped interventions, such as combining bone marrow aspirate concentrate (BMAC), platelet-rich plasma (PRP), and bone marrow blood cells (BBC), as CellTherapy, and combined interventions using Alendronate sodium tablets or Chinese herbal Fufang Xian Ling Gu Bao as injectable med. The incorporation of both direct and indirect evidence enabled a comprehensive comparison of these diverse interventions. Indirect comparisons played a critical role in our study since most treatment methods were not directly compared in the RCTs. The main findings showed that CellTherapy, ESWT+InjectableMed, InjectableMed alone, and ESWT were more effective than CD+PTRI, CD+BG+CellTherapy, and CD+BG in terms of the VAS pain score. Furthermore, CD+BG+CellTherapy was more effective than CD+BG. In terms of the duration of efficacy, CellTherapy, ESWT, ESWT+InjectableMed, and InjectableMed had better short-term effects on pain relief at 12 months than CD+PTRI and CD+BG. Beyond 12 months, ESWT was more effective in relieving pain than CD+BG. Pairwise meta-analysis was conducted on the comparison between CD+CellTherapy and CD monotherapy in two articles as it could not be linked to the aforementioned interventions, and whether to combine CellTherapy with CD treatment is also worth exploring when using CD therapy. The results exhibited no significant statistical difference in the impact on VAS between CD+CellTherapy and CD alone, and the effects of both groups were comparable. This may be due to the limited number of studies included and insufficient supporting evidence.

Based on the SUCRA probability ranking, ESWT+InjectableMed has the highest efficacy rate (87%) for treatment outcomes within 12 months, followed by CellTherapy (78.3%), InjectableMed (77.7%), ESWT (56.6%), CD+BG+CellTherapy (31.1%), CD+PTRI (15.7%), and CD+BG (3.5%). For long-term effects, ESWT was found to be the most effective, followed by CD+BG+CellTherapy and CD+BG. These findings suggest that non-surgical treatments (CellTherapy, ESWT+InjectableMed, InjectableMed, and ESWT) are more effective than surgical treatments (CD+PTRI, CD+BG+CellTherapy, and CD+BG) in treating non-traumatic ONFH pain. These findings are consistent with a review published by Wojciech Konarski et al. in 2022 (26), which also found pain relief to be the main observed effect.

According to Russo et al. in 2015 (27), the mechanism of action for ESWT is believed to be the stimulation of osteoblast activity, which increases bone density in the pelvic area. This treatment was found to be more effective than transplantation and core decompression in the early stages of the disease. In a single-arm study conducted by Sanjay Agarwala et al. in 2019 (28), proved to be effective in providing pain relief and delaying radiological progression, further emphasizing the benefits of combining drug therapy with ESWT for optimal clinical outcomes. The combination of oral alendronate and intravenous zoledronic acid was found to be a practical solution for non-traumatic ONFH. The combination therapy not only provided pain relief but also resulted in long-term delayed radiological progression, thereby avoiding the necessity for surgery. The therapy was well-tolerated, and 94.4% of patients showed good clinical improvement in the early stages of the disease. Combining drug therapy with ESWT was found to result in a more optimal clinical improvement effect.

The study utilized HHS scores to directly and indirectly compare different intervention measures for non-traumatic femoral head necrosis. However, no significant statistical differences were noticed between the intervention measures. Thus, the SUCRA probability ranking was used to determine treatment effects. CellTherapy (77%) was found to have the best effect, followed by ESWTInjectableMed (72.2%), ESWT (58.3%), InjectableMed (50%), CD+PTRI (31.4%), and CD+BG (11%). The results were consistent with the VAS, indicating that CellTherapy and ESWT, with or without medication, are effective in treating patients with non-traumatic femoral head necrosis. Two studies on intervention measures of CD+CellTherapy and CD monotherapy were not part of the network with other measures; therefore, pairwise meta-analysis was conducted. The results showed that the short-term effect of CD combined with CellTherapy was better than that of CD alone. However, in the long-term effect, the two groups were found to be equivalent.

It is important to consider that pain and impaired functions in patients with non-traumatic femoral head necrosis may arise not only from the disease itself but also from the treatment process. Surgical treatments with trauma have been shown to be less effective than non-surgical treatments based on both VAS and HHS indicators. This is likely due to the additional pain and physical limitations caused by the damage to body tissues during surgical interventions. In evaluating joint function recovery, pairwise meta-analysis of the WOMAC and Lequesne scores revealed similar effects between CD+BG+CellTherapy and CD+BG. This highlights that while surgical interventions may lead to temporary improvement, non-surgical treatments tend to offer better overall outcomes in terms of pain relief and function recovery.

This meta-analysis, which is based on RCTs, represents the first comprehensive evaluation of the efficacy of multiple interventions for the treatment of ONFH. The study employed both pairwise and network meta-analysis techniques to analyze the effectiveness of nine interventions, including CellTherapy, InjectableMed, ESWT, ESWT+InjectableMed, CD+PTRI, CD+BG+CellTherapy, CD+BG, and CD alone. Stratifying the interventions based on their short-term and long-term effects provides a clearer understanding of their relative efficacy.

The present study recommends the use of ESWT+InjectableMed for short-term or more acute/short-term patients, whereas ESWT is suggested for long-term patients suffering from osteonecrosis. Our findings offer significant insights into the management of this condition and call for greater cooperation between physicians and patients to preserve the femoral head or hip joint. However, several limitations should be acknowledged. Firstly, the strength of the evidence is insufficient due to the limited number of literature sources included. The network meta-analysis heavily relied on indirect evidence, resulting in a lack of robust direct pairwise comparisons. This limitation poses significant challenges to the validity and reliability of the conclusions drawn, as direct comparisons between treatments provide more definitive insights. Secondly, there was an absence of organized categorization and thorough examination of other crucial elements such as disease staging, lesion size, and the distinction between unilateral and bilateral illness. These factors are crucial for comprehending the variety in treatment outcomes, and neglecting them can result in biased conclusions. Enhancing the clarity and application of the findings would be achieved by using a more precise and systematic approach in categorizing these factors. Thirdly, we did not analyze the specific cell types used in CellTherapy. This omission is critical because different cell types can have varying therapeutic effects and mechanisms of action, which in turn can influence the overall efficacy and safety profile of the treatment. Additionally, drug therapy in our study did not differentiate between Western and Chinese medicine. This lack of distinction is important because it introduces potential heterogeneity, given the differences in pharmacological mechanisms and therapeutic principles between these two medical traditions. Addressing these aspects would provide a more nuanced and accurate interpretation of the data, thereby improving the overall quality and relevance of the study outcomes.

During the literature search, we observed that most RCT studies were published in recent years, highlighting the growing attention given by the academic community towards the treatment of ONFH. Future research with larger sample sizes and multi-center designs are warranted to further explore the effects of different interventions for the treatment of this condition, so as to provide more scientifically rigorous and reliable treatment regimens for patients suffering from ONFH.

## Conclusion

In conclusion, the available evidence suggests that CellTherapy, non-surgical ESWT combined with drugs or CellTherapy, are the most effective interventions for the treatment of ONFH. Specifically, CellTherapy consistently shows significant improvement in VAS pain scores and HHS scores, making it a leading treatment option. When CD+BG surgery is necessary, combining it with CellTherapy is recommended, as this approach is more effective than CD+BG alone. The effect of CD+PTRI is between the two interventions. For short-term or acute/short-course patients, ESWT+InjectableMed is recommended, while ESWT is recommended for long-term cases. However, due to the limitations of this study, more high-quality clinical trials with larger sample sizes and multicenter designs are needed to verify our findings and provide more scientifically sound treatment options for patients with ONFH.

## Data Availability

The original contributions presented in the study are included in the article/**Supplementary Material**. Further inquiries can be directed to the corresponding author.
