# Supplementary material for: Effectiveness of various interventions for non-traumatic osteonecrosis: a pairwise and network meta-analysis
Source: Front Endocrinol (Lausanne). 2024 Aug 21;15:1428125. doi: 10.3389/fendo.2024.1428125 (PMC11371630; doi:10.3389/fendo.2024.1428125)
Supplement: Supplementary file 1 [file DataSheet1.docx]

**Appendix 1 Search strategy**

**Pubmed**

| Search number | Query | Results |
| --- | --- | --- |
| #1 | "Sarcopenia"[Mesh] | 7526 |
| #2 | (Sarcopenia[Title/Abstract]) OR (Sarcopenias[Title/Abstract]) | 12,923 |
| #3 | ("Sarcopenia"[Mesh]) OR ((Sarcopenia[Title/Abstract]) OR (Sarcopenias[Title/Abstract])) | 13,826 |
| #4 | "Resistance Training"[Mesh] | 11,205 |
| #5 | (((((((((((((((((((((((Resistance Training[Title/Abstract]) OR (Training, Resistance[Title/Abstract])) OR (Strength Training[Title/Abstract])) OR (Training, Strength[Title/Abstract])) OR (Weight-Lifting Strengthening Program[Title/Abstract])) OR (Strengthening Program, Weight-Lifting[Title/Abstract])) OR (Strengthening Programs, Weight-Lifting[Title/Abstract])) OR (Weight Lifting Strengthening Program[Title/Abstract])) OR (Weight-Lifting Strengthening Programs[Title/Abstract])) OR (Weight-Lifting Exercise Program[Title/Abstract])) OR (Exercise Program, Weight-Lifting[Title/Abstract])) OR (Exercise Programs, Weight-Lifting[Title/Abstract])) OR (Weight Lifting Exercise Program[Title/Abstract])) OR (Weight-Lifting Exercise Programs[Title/Abstract])) OR (Weight-Bearing Strengthening Program[Title/Abstract])) OR (Strengthening Program, Weight-Bearing[Title/Abstract])) OR (Strengthening Programs, Weight-Bearing[Title/Abstract])) OR (Weight Bearing Strengthening Program[Title/Abstract])) OR (Weight-Bearing Strengthening Programs[Title/Abstract])) OR (Weight-Bearing Exercise Program[Title/Abstract])) OR (Exercise Program, Weight-Bearing[Title/Abstract])) OR (Exercise Programs, Weight-Bearing[Title/Abstract])) OR (Weight Bearing Exercise Program[Title/Abstract])) OR (Weight-Bearing Exercise Programs[Title/Abstract]) | 15,792 |

**2.Cochrane**

| Search number | Query | Results |
| --- | --- | --- |
| #1 | MeSH descriptor: [Sarcopenia] explode all trees | 597 |
| #2 | (Sarcopenia):ti,ab,kw OR (Sarcopenias):ti,ab,kw | 1705 |
| #3 | MeSH descriptor: [Resistance Training] explode all trees | 4108 |
| #4 | (Resistance Training):ti,ab,kw OR (Training, Resistance):ti,ab,kw OR (Strength Training):ti,ab,kw OR (Training, Strength):ti,ab,kw OR (Weight-Lifting Strengthening Program):ti,ab,kw | 23122 |
| #5 | (Strengthening Program, Weight-Lifting):ti,ab,kw OR (Strengthening Programs, Weight-Lifting):ti,ab,kw OR (Weight Lifting Strengthening Program):ti,ab,kw OR (Weight-Lifting Strengthening Programs):ti,ab,kw OR (Weight-Lifting Exercise Program):ti,ab,kw | 266 |
| #6 | (Exercise Program, Weight-Lifting):ti,ab,kw OR (Exercise Programs, Weight-Lifting):ti,ab,kw OR (Weight Lifting Exercise Program):ti,ab,kw OR (Weight-Lifting Exercise Programs):ti,ab,kw OR (Weight-Bearing Strengthening Program):ti,ab,kw | 416 |
| #7 | (Strengthening Program, Weight-Bearing):ti,ab,kw OR (Strengthening Programs, Weight-Bearing):ti,ab,kw OR (Weight Bearing Strengthening Program):ti,ab,kw OR (Weight-Bearing Strengthening Programs):ti,ab,kw OR (Weight-Bearing Exercise Program):ti,ab,kw | 434 |
| #8 | (Exercise Program, Weight-Bearing):ti,ab,kw OR (Exercise Programs, Weight-Bearing):ti,ab,kw OR (Weight Bearing Exercise Program):ti,ab,kw OR (Weight-Bearing Exercise Programs):ti,ab,kw | 454 |
| #9 | MeSH descriptor: [Nutrition Therapy] explode all trees | 10290 |
| #10 | (Nutrition Therapy):ti,ab,kw OR (Therapy, Nutrition):ti,ab,kw OR (Medical Nutrition Therapy):ti,ab,kw OR (Nutrition Therapy, Medical):ti,ab,kw OR (Therapy, Medical Nutrition):ti,ab,kw | 8798 |
| #11 | #1 OR #2 | 1705 |
| #12 | #3 OR #4 OR #5 OR #6 OR #7 OR #8 | 23434 |
| #13 | #9 OR #10 | 16703 |
| #14 | #11 AND #12 AND #13 | 29 |

**3.Embase**

| Search number | Query | Results |
| --- | --- | --- |
| #1 | 'sarcopenia'/exp | 16038 |
| #2 | sarcopenia:ti,ab,kw OR sarcopenias:ti,ab,kw | 19850 |
| #3 | 'resistance training'/exp | 23695 |
| #4 | 'training, resistance':ti,ab,kw OR 'resistance training':ti,ab,kw OR 'training, strength':ti,ab,kw OR 'weight-lifting strengthening program':ti,ab,kw OR 'strengthening program, weight-lifting':ti,ab,kw OR 'strengthening programs, weight-lifting':ti,ab,kw OR 'weight lifting strengthening program':ti,ab,kw OR 'weight-lifting strengthening programs':ti,ab,kw OR 'weight-lifting exercise program':ti,ab,kw OR 'exercise program, weight-lifting':ti,ab,kw OR 'exercise programs, weight-lifting':ti,ab,kw OR 'weight lifting exercise program':ti,ab,kw OR 'weight-lifting exercise programs':ti,ab,kw OR 'weight-bearing strengthening program':ti,ab,kw OR 'strengthening program, weight-bearing':ti,ab,kw OR 'strengthening programs, weight-bearing':ti,ab,kw OR 'weight bearing strengthening program':ti,ab,kw OR 'weight-bearing strengthening programs':ti,ab,kw OR 'weight-bearing exercise program':ti,ab,kw OR 'exercise program, weight-bearing':ti,ab,kw OR 'exercise programs, weight-bearing':ti,ab,kw OR 'weight bearing exercise program':ti,ab,kw OR 'weight-bearing exercise programs':ti,ab,kw | 12603 |
| #5 | 'diet therapy'/exp | 401329 |
| #6 | 'nutrition therapy':ti,ab,kw OR 'therapy, nutrition':ti,ab,kw OR 'medical nutrition therapy':ti,ab,kw OR 'nutrition therapy, medical':ti,ab,kw OR 'therapy, medical nutrition':ti,ab,kw | 3549 |
| #7 | #1 OR #2 | 22080 |
| #8 | #3 OR #4 | 27282 |
| #9 | #5 OR #6 | 402425 |
| #10 | #7 AND #8 AND #9 | 339 |

**4.Web of science**

| Search number | Query | Results |
| --- | --- | --- |
| #1 | Sarcopenia (Topic) or Sarcopenias (Topic) | 23908 |
| #2 | Nutrition Therapy (Topic) or Therapy, Nutrition (Topic) or Medical Nutrition Therapy (Topic) or Nutrition Therapy, Medical (Topic) or Therapy, Medical Nutrition (Topic) 486320 | 486320 |
| #3 | Resistance Training (Topic) or Training, Resistance (Topic) or Strength Training (Topic) or Training, Strength (Topic) or Weight-Lifting Strengthening Program (Topic) or Strengthening Program, Weight-Lifting (Topic) or Strengthening Programs, Weight-Lifting (Topic) or Weight Lifting Strengthening Program (Topic) or Weight-Lifting Strengthening Programs (Topic) or Weight-Lifting Exercise Program (Topic) or Exercise Program, Weight-Lifting (Topic) or Exercise Programs, Weight-Lifting (Topic) or Weight Lifting Exercise Program (Topic) or Weight-Lifting Exercise Programs (Topic) or Weight-Bearing Strengthening Program (Topic) or Strengthening Program, Weight-Bearing (Topic) or Strengthening Programs, Weight-Bearing (Topic) or Weight Bearing Strengthening Program (Topic) or Weight-Bearing Strengthening Programs (Topic) or Weight-Bearing Exercise Program (Topic) or Exercise Program, Weight-Bearing (Topic) or Exercise Programs, Weight-Bearing (Topic) or Weight Bearing Exercise Program (Topic) or Weight-Bearing Exerc ise Programs (Topic) | 148313 |
| #4 | #1 AND #2 AND #3 | 266 |
